# Identification of *Prdm* genes in human corneal endothelium

**DOI:** 10.1016/j.exer.2017.02.009

**Published:** 2017-06

**Authors:** Kostadin Rolev, Dominic G. O'Donovan, Christiana Georgiou, Madhavan S. Rajan, Alexandra Chittka

**Affiliations:** a Anglia Ruskin University, Department of Biomedical and Forensic Sciences and the Vision & Eye Research Unit, Cambridge CB1 1PT, United Kingdom; b Department of Ophthalmology, Cambridge University Hospitals, Hills Road, Cambridge, Cambridgeshire CB2 0QQ, United Kingdom; c The Wolfson Institute for Biomedical Research, Division of Medicine, UCL, Gower St, London WC1E 6BT, United Kingdom; d Dept. of Histopathology, Cambridge University Hospitals, Hills Road, Cambridge, Cambridgeshire CB2 0QQ, United Kingdom

**Keywords:** Prdm (Prdm refers to the protein, *Prdm* refers to mRNA), Corneal endothelial cells, Cornea, CEC, corneal endothelial cells, Prdm, Positive regulatory domain, SET, suppressor of variegation 3–9, enhancer of zeste, trithorax, DAB, 3,3'-diaminobenzidine

## Abstract

Corneal endothelial cells (CECs) are essential for maintaining corneal stromal hydration and ensuring its transparency, which is necessary for normal vision. Dysfunction of CECs leads to stromal decompensation, loss of transparency and corneal blindness. Corneal endothelium has low proliferative potential compared to surface epithelial cells leading to poor regeneration of CEC following injury. Additionally, the tissue exhibits age related decline in endothelial cell density with re-organisation of the cell layer, but no regeneration. The mechanisms which control proliferation and differentiation of neural crest derived CEC progenitors are yet to be clearly elucidated.

Prdm (Positive regulatory domain) family of transcriptional regulators and chromatin modifiers are important for driving differentiation of a variety of cellular types. Many Prdm proteins are expressed in specific precursor cell populations and are necessary for their progression to a fully differentiated phenotype. In the present work, we sought to identify members of the *Prdm* gene family which are specifically expressed in human (h) CECs with a view to begin addressing their potential roles in CEC biology, focussing especially on *Prdm 4* and *5* genes. By performing semi-quantitative reverse transcription coupled to PCR amplification we found that in addition to *Prdm4* and *Prdm5*, *Prdm2* and *Prdm10* genes are expressed in hCECs. We further found that cultured primary hCECs or immortalised HCEC-12 cells express all of the *Prdm* genes found in CECs, but also express additional *Prdm* transcripts. This difference is most pronounced between *Prdm* gene expression patterns of CECs isolated from healthy human corneas and immortalised HCEC-12 cells. We further investigated Prdm 4 and Prdm 5 protein expression in cultured primary hCECs and HCEC-12 cells as well as in a human cadaveric whole cornea. Both Prdm 4 and Prdm 5 are expressed in human corneal endothelium, primary hCECs and in HCECs-12 cells, characterised by expression of the Na^+^/K^+^-ATPase. We observed that both proteins exhibit cytosolic (intracellular, but non-nuclear and distinct from extracellular fluid) as well as nuclear localisation within the endothelial layer, with Prdm 5 being more concentrated in the nuclei of the endothelial cells than Prdm 4. Thus, our work identifies novel *Prdm* genes specifically expressed in corneal endothelial cells which may be important in the control of CEC differentiation and proliferation.

## Highlights

• The analysis of *Prdm* (Positive regulatory domain) family of transcriptional regulators and chromatin modifying genes in corneal endothelial cells (CECs) was undertaken.
• *Prdm 2*, *4*, *5* and *10* genes were found to be expressed in human CECs.
• Prdm 4 and 5 protein expression was characterised in CECs, ex vivo cultured corneal endothelial cells, and the intact cornea.

## 1 Introduction

Corneal endothelial cells (CECs) form a monolayer of highly specialised cells found at the most posterior part of the cornea, which maintain the stroma at the normal physiological level of hydration at ca 78%. Loss of the endothelial cells leads to the swelling of cornea and loss of corneal transparency (Eghrari et al., 2015, Parekh et al., 2016). Currently, the treatment of such decompensated corneas relies on human cadaveric corneal donor transplantation with associated problems of immune rejection and lack of immediate availability of donor tissue (Parekh et al., 2016). Therefore, understanding the mechanisms which control hCEC proliferation and differentiation from their precursors or from stem cells/induced pluripotent stem cells (iPSCs) via re-programming is highly desirable in order to begin developing rational strategies for generating hCECs for therapeutic purpose.

Prdm proteins are a family of structurally related transcriptional regulators, characterised by the presence of at least two distinct domains, the zinc fingers and the PR/SET (named after the *Drosophila* transcription factors Suppressor of variegation 3–9, Enhancer of Zeste and Trithorax (SET) and Positive regulatory domain I-binding factor 1/Retinoblastoma protein-interacting zinc finger gene 1 (PR) motifs originally identified in Prdm1 and Prdm2, respectively) domains, similar to, but distinct from the SET domains of histone lysine methyltransferases (Fog et al., 2012, Hohenauer and Moore, 2012). Many of the Prdm proteins are instrumental in controlling differentiation of a variety of cell types (Fog et al., 2012, Hohenauer and Moore, 2012, Di Zazzo et al., 2013, Zannino and Sagerström, 2015). For example, Prdm 1 is involved in the specification of primordial germ cells, forelimb patterning, and reprogramming of intestinal enterocytes (Ohinata et al., 2005, Bikoff et al., 2009, Harper et al., 2011). Prdm 16 controls brown fat cell identity as well as haematopoiesis and cardiac development (Zhang et al., 2011, Chi and Cohen, 2016). Prdm 8 is required for rod bipolar and type 2 OFF-cone bipolar cell survival and amacrine subtype identity and its absence resembles the incomplete form of human congenital stationary night blindness (CSNB) (Jung et al., 2015). Importantly, Prdm 5 regulates expression of extracellular matrix components and mutations in *Prdm 5* gene lead to the brittle cornea syndrome (BCS) (Burkitt Wright et al., 2011, Aldahmesh et al., 2012, Micheal et al., 2016a). Prdm 5 is the only Prdm protein with a well-documented and characterised physiological role in corneal cell biology. Intriguingly, Prdm 4 appears to control some aspects of corneal biology as evidenced by the initial characterisation of the mutant mice where *Prdm 4* gene is deleted, which show abnormalities in corneal morphology, thus making it a potentially novel candidate gene that may be useful for therapeutic purposes (MRC Harwell web reference).

Given the importance of Prdm proteins in controlling cell fate decisions during development, we reasoned that some of them may regulate various aspects of CEC proliferation and differentiation. To date, no systematic analysis of expression of these genes in corneal endothelial cells has been undertaken. Here, we present the first analysis of expression of all human *Prdm* genes in hCECs and whole cadaveric cornea with special focus on the expression of *Prdm 4* and *5* genes because of their documented involvement in corneal function. Further, we investigate the expression of *Prdm* genes in cultured primary hCECs and an immortalised cell line, HCEC-12 and find that both show overlapping, but distinct expression of *Prdm* genes as compared to CECs or the whole human cornea. Isolated Descemet's membranes together with the attached CECs express *Prdm 2, 4, 5, 10* and very weakly *Prdm 11* mRNA. Within the whole cornea, we detected *Prdm 1, 2, 4, 5* and *10* mRNA expression. Moreover, we demonstrate that two of the selected Prdm proteins, Prdm 4 and Prdm 5, are expressed in both primary hCECs and HCEC-12 cells. Both Prdm 4 and 5 are found in the endothelial cells of the whole cornea. Our results pave the way for further investigations into the roles of these *Prdm* genes in CEC proliferation, maintenance and differentiation which may be useful for generating CECs for cell transplantation.

## 2 Material and methods

### 2.1 RNA extraction and Reverse transcription-PCR (RT-PCR) analysis of *Prdm* gene expression

RNA was extracted from whole human donor corneas, Descemet's membrane (DM)/Endothelium complex, primary cultured human corneal endothelial cells (hCECs) and from cultured HCEC-12 cells. To isolate total RNA from the normal cornea, Trizol^®^ reagent (Invitrogen/ThermoFisher Scientific) was used according to manufacturer's instructions. Human donor corneas were obtained from Bristol and Manchester Eye Banks, UK, with research consent. To isolate total RNA from the DM/Endothelium complex, hCEC and HCEC-12 cells, we used micro-prep RNA extraction kit from Agilent Technologies. A total of 5 × 10^5^ HCEC-12 and primary hCECs were harvested at passage six and two, respectively, following seven days of culturing, for RNA extraction.

SuperScript^®^ II Reverse Transcriptase was used to perform RT according to the manufacturer's instructions using 1 μg of total RNA and random hexamer primer mix (Invitrogen/ThermoFisher Scientific). Subsequently, 1 μl of cDNA was used for PCR amplification with gene-specific primers. Control reactions were performed without the addition of reverse transcriptase in the reaction mix. Cycling for PCR amplification was performed using the following programme: 94 °C for 2 min followed by 40 cycles of 94 °C for 30 s, 59 °C for 30 s, 72 °C for 30 s, and a final extension step at 72 °C for 5 min. PCR products were separated using 2% agarose gel electrophoresis.

The following gene-specific primers were used: Prdm1F 5′ CAGTTCCTAAGAACGCCAACAGG 3’; Prdm1R 5′ GTGCTGGATTCACATAGCGCATC 3’; Prdm2F 5′ TTGGGCTTGCTCAGGAGAAGAG 3’; Prdm2R 5′ GCTGCTATCTCAGGGTTGTCTTC 3’; Prdm3(MDS)F 5′ CCTTATGTGGGAGAGCAGAGGT 3’; Prdm3(MDS)R 5′ GAAGGCTATTCCTACGTCTGAGC 3’; Prdm3(MECOM)F 5′ CCTGCTTCAGATGGTTCCTTGC 3’; Prdm3(MECOM)R 5′ GGTGAAACAAGAATCCTGGAGAAG 3’; Prdm4F 5′ CCAAAGCAGCTTGTTCTCCGTC 3’; Prdm4R 5′ TAGAGGTCCAAAGCAAGTCCGC 3’; Prdm5F 5′ ACTCTGAGGAGAGACCGTTCCA 3’; Prdm5R 5′ AGCATCGCAGTGATGGCACTTG 3’; Prdm6F 5′ GTGAGGAACACGCAGCATCTCT 3’; Prdm6R 5′ TTCTCCGCAGTGCCTTGCACAT 3’; Prdm7F 5′ AGACGAAGAGGCAGCCAACAGT 3’; Prdm7R 5′ GCCACCAGGTTCTGCTCTTCAT 3’; Prdm8F 5′ CTGTGTCCTGAGCCATACTTCC 3’; Prdm8R 5′ CCTTCTGAGGAACCATTTGCTGC 3’; Prdm9F 5′ ACGAAGAGGCAGCCAACAATGG 3’; Prdm9R 5′ GCCACCAGGTTCTGCTCTTCAT 3’; Prdm10F 5′ CCGCAAAGACTTCCTGTGTTCC 3’; Prdm10R 5′ GCTGATGCGGTCGGCTTTCTTG 3’; Prdm11F 5′ CCAGGAAACCATTCACCGCAAC 3’; Prdm11R 5′ CCTCAGGTCTTCTGGGTTATCC 3’; Prdm12F 5′ TGCACGTAACGAACAGGAGCAG 3’; Prdm12R 5′ GTGAGTTTCCGTACCACACCAG 3’; Prdm13F 5′ CTAACTCCTTGGCTCAGTGGTTC 3’; Prdm13R 5′ CAGTACCAGCAGATGTAGCGCT 3’; Prdm14F 5′ CCTTGTGTGGTATGGAGACTGC 3’; Prdm14R 5′ CTTTCACATCTGTAGCCTTCTGC 3’; Prdm15F 5′ GGCACTTGTGAGAAGACCTTCC 3’; Prdm15R 5′ GAGGTTGCTGTTGGTGGAGAAG 3’; Prdm16F 5′ CAGCCAATCTCACCAGACACCT 3’; Prdm16R 5′ GTGGCACTTGAAAGGCTTCTCC 3’.

### 2.2 Primary and immortalised cell culture

Primary hCEC culture from peripheral DM with endothelial cells (female, 64 years old, cadaveric donor cornea) was initiated via primary explant culture. DM was cut into small pieces with fine scissors and placed into a T25 cell culture flask. Cells were initially maintained in 1 ml basal medium OptiMEM-I supplemented with 8% Fetal Bovine Serum (FBS), 1% L-Glutamine, 1% penicillin/streptomycin (all from Invitrogen/ThermoFisher Scientific). Small amount of medium was used in order to allow the pieces of the peripheral DM to come in contact with the bottom surface of the flask and to promote cell outgrowth into the flask. The culture medium was not exchanged until primary cell outgrowth occurred, usually 7–14 days after culture initiation. After that, the culture medium was exchanged every 2nd day until the culture reached confluence. After reaching confluence the primary culture was sub-cultured using 0.05% trypsin/EDTA.

HCEC-12 cells, purchased from The Leibniz Institute DSMZ (German Collection of Microorganisms and Cell Cultures; Deutsche Sammlung von Mikroorganismen und Zellkulturen, Catalogue number ACC 646), were cultured using F99 (mixture of Ham's F12 and Medium 199 (ThermoFisher Scientific) in 1:1 ratio) medium supplemented with 10% FBS. The medium was changed every 2 days. The cells were seeded out at the density of 50 cells/mm^2^. Confluent cultures were split 1:2 to 1:4 every 4–5 days using 0.05% trypsin/EDTA. All cell cultures were kept in a humidified Heracell ™ incubator at 37 °C, 5% CO_2_, 95–98%.

### 2.3 Immunofluorescent staining and laser scanning confocal microscopy of cells

HCEC-12 and primary hCECs were cultured in glass chamber slides, coated with 0.1 mg/ml poly-D-lysine (PDL) (Sigma Aldrich), at 5000 cells/cm^2^ density for 24 h prior to fixing and processing for immunocytochemistry. The cells were fixed in 4% paraformaldehyde for 10 min at room temperature and permeabilised using blocking solution (10% normal goat serum (Invitrogen/ThermoFisher Scientific) in PBS) supplemented with 0.1% v/v Triton X-100 for 20 min at room temperature. Following permiabilisation, cells were incubated in blocking solution for one hour at room temperature. Primary antibodies were added to the fixed cells in blocking solution overnight at 4 °C, followed by the incubation with appropriate secondary antibodies. The following primary antibodies were used: rabbit anti-*Prdm*4 antibody, Abcam (ab156867), rabbit anti-*Prdm*5 antibody, Abcam (ab79016) and rabbit anti-*Prdm*5, Abcam antibody (ab84572), all at 1:500 dilution, and mouse monoclonal anti-alpha 1 Na+/K + - ATPase antibody, Abcam (ab7671) at a 1:100 dilution. Secondary goat anti-rabbit and anti-mouse antibodies conjugated to Alexa 488 or Alexa 568 (Invitrogen/ThermoFisher Scientific) were used at 1:1000 dilution together with Hoechst DNA stain prior to mounting immunolabelled cells in DAKO mounting medium. Immunolabelled cells were visualized using the Leica Microsystems CMS confocal microscope.

### 2.4 Immunohistochemical labelling of human cornea

Cornea were fixed in formalin and embedded in paraffin for processing using LEICA Bond-III automated system with LEICA Bond Polymer Refine Detection kit (DS9800) according to the manufacturer's instruction. Bond Polymer Refine Detection kit is a biotin-free, polymeric horseradish peroxidase (HRP)-linker antibody conjugate system for the detection of tissue-bound mouse and rabbit IgG's. All sections were subjected to antigen heat retrieval at 100 °C for 20 min prior to staining procedure using either Epitope retrieval solution 1 (pH 6.0) (AR9961) for anti-*Prdm*4 antibodies or Epitope retrieval solution 2 (pH9.0) (AR9640) for anti-*Prdm*5 antibodies. Both antibodies were used at 1:500 dilution. The staining programme used was as follows: 1) peroxide block - 5min, 2) primary antibody was applied for 30 min, 3) polymer HRP was added for 8 min, 4) mixed DAB was added for 10 min. Sections were counterstained with haematoxylin for 5 min to visualise nuclei.

## 3 Results

### 3.1 Expression of *Prdm* genes in healthy human cornea and endothelial cell/Descemet membrane complex

To begin our investigations we initially utilized cadaveric whole human cornea to assess expression profiles of all human *Prdm* genes. The corneas obtained were from deceased 64 year old female, 69 year old male and 87 year old male.

**Fig. 1:**
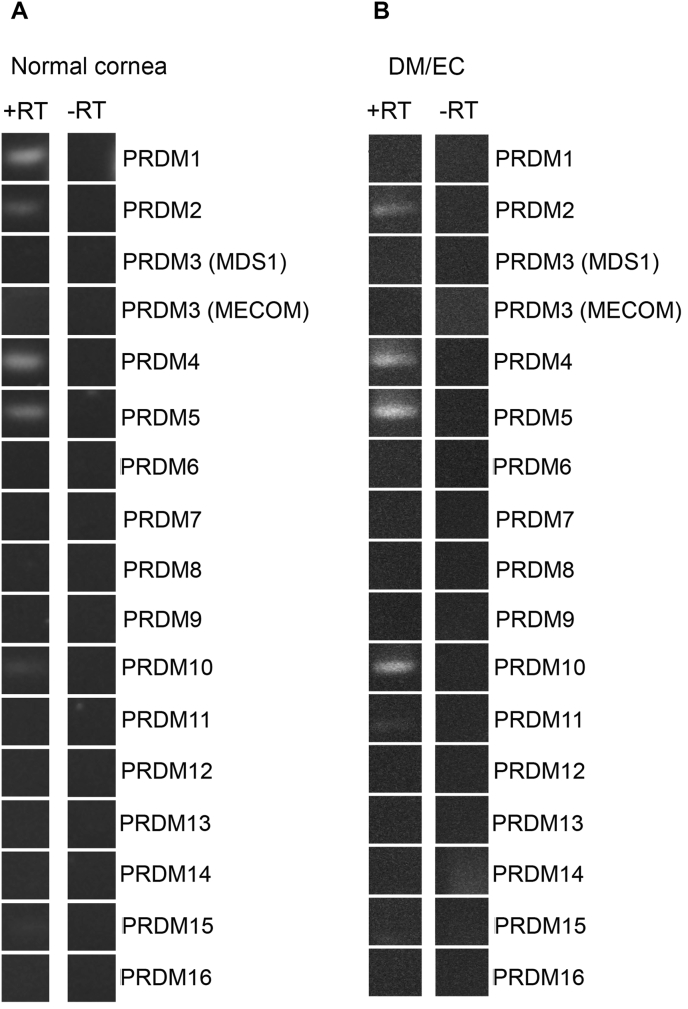
Expression of *Prdm* genes mRNAs in intact cornea and Descemet membrane/corneal endothelial cells. A) *Prdm* gene mRNA assessed by RT-PCR with gene specific primers in intact cornea and, B) in the DM/CECs. *Prdm 1* expression is lost in the DM/CECs and a very weak *Prdm 11* expression becomes detectable.

To this end, total RNA isolated from the cornea were reverse transcribed and the expression of *Prdm* genes was assessed using gene-specific primers. We detected expression of five *Prdm* mRNAs out of a total of 17 primer combinations tested. *Prdm 1, 4* and *5* were expressed at relatively high levels, while *Prdm 2* and *10* showed moderate to low expression levels in total normal human cornea (Fig. 1A). Interestingly, when endothelial cells/Descemet membrane complex alone were used as a source of total RNA, expression of *Prdm 1* was not detectable and we also detected a very weak expression of *Prdm 11* in the sample (Fig. 1B). Taken together, our observations suggest that normal human cornea express *Prdm 1, 2, 4, 5* and *10* as part of their normal transcriptional profile, while Descemet Membrane with corneal endothelial cells (DM/CECs) express *Prdm 2, 4, 5, 10* and very weakly *11* (summarized in Table 1).

**Table 1:** **Summary of *Prdm* gene expression in normal cornea, DM/CECs and primary and immortalised CECs.** + denotes expression of mRNA for the respective gene; +/− denotes weak expression of mRNA for a specific gene.

	Normal Cornea	DM/CECs	Primary HCEC	Immortalised HCECs
*Prdm1*	+		+	+
*Prdm2*	+	+	+	+
*Prdm3 (MDS1)*				
*Prdm3 (MECOM)*				+
*Prdm4*	+	+	+	+
*Prdm5*	+	+	+	+
*Prdm6*				+
*Prdm7*				
*Prdm8*			+	+
*Prdm9*				
*Prdm10*	+	+	+	+
*Prdm11*		+/−		+
*Prdm12*				
*Prdm13*				
*Prdm14*				
*Prdm15*				
*Prdm16*				+

### 3.2 Expression of *Prdm* genes in primary and immortalised hCECs

**Fig. 2:**
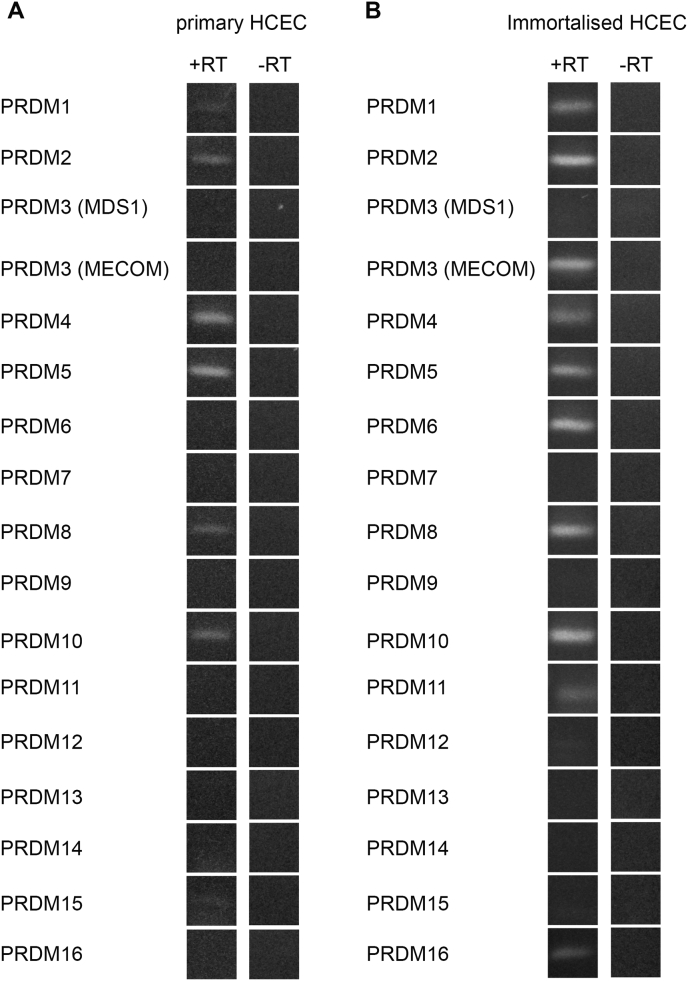
Expression of *Prdm* genes mRNAs in primary hCECs and immortalised HCEC-12 cell line. A) *Prdm* genes expressed in primary hCECs harvested after two passages of the culture and, B) *Prdm* genes expressed in primary HCEC-12 cell line harvested after six passages of the culture. Note that expression profile of *Prdm* genes is much closer to the one observed in intact cornea in primary hCECs than in HCEC-12 immortalised cells.

We characterised the expression profiles of *Prdm* genes in primary hCECs by performing RT-PCR as outlined in section 3.1. We detected expression of *Prdm 1, 2, 4, 5* and *10* in primary hCECs as was the case for healthy normal cornea (Fig. 2A). However, we also detected *Prdm 8* expression in our primary hCEC culture, which was not detectable in whole human corneas. Therefore, while the *Prdm* gene expression profile of primary hCECs is similar to that of the normal human cornea, it is not identical to it (summarized in Table 1).

We utilized the cell line, HCEC-12 and investigated the expression profile of *Prdm* genes in these cells. Whilst all *Prdm* genes which are expressed in normal human cornea and DM/CECs are also found in HCEC-12 cell line, there are several additional transcripts found in these cells (Fig. 2B). Thus, in addition to *Prdm 1, 2, 4, 5* and *10*, we also detected expression of the following *Prdm* genes in HCEC-12 cells: *Prdm 3* (MECOM isoform), *Prdm 6, 8, 11* and *16* (summarized in Table 1). Taken together, these observations suggest that HCEC-12 cell line has a distinctly different transcriptional profile from healthy human corneas.

### 3.3 Expression of *Prdm* 4 and *Prdm* 5 proteins in HCEC-12 cell line and primary hCECs

**Fig. 3:**
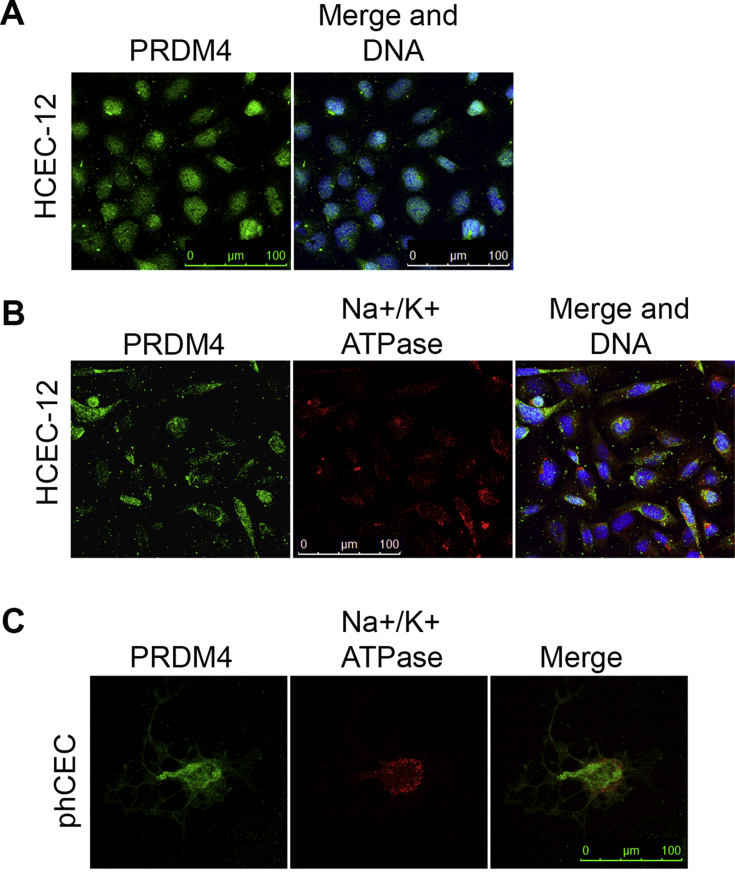
Prdm 4 protein is expressed in HCEC-12 cells and primary hCECs. A) Fluorescent immunolabelling of HCEC-12 cells reveals that Prdm 4 protein (green) is localised to the nucleus and the cytosol of immortalised HCEC-12 cells. B) HCEC-12 cells expressing Prdm 4 proteins also express CEC marker, Na^+^/K^+^-ATPase (red), which is localised predominantly to the membrane of the cells. C) Prdm 4 protein (green) is expressed in primary hCECs (phCEC) together with the Na^+^/K^+^-ATPase (red). Note that Prdm 4 protein is mainly detected in the cytosol in these cells with the Na^+^/K^+^-ATPase being confined to the membrane predominantly. Nuclei are counterstained with Hoechst (blue). (For interpretation of the references to colour in this figure legend, the reader is referred to the web version of this article.)

Given the importance of Prdm 5 and 4 in corneal biology (Burkitt Wright et al., 2011, Aldahmesh et al., 2012, Porter et al., 2015a, Micheal et al., 2016a, Micheal et al., 2016b) and (MRC Harwell web reference), we next focused our attention on the expression of these two proteins in cultured hCECs. Immunostaining of HCEC-12 cells to detect Prdm 4 expression revealed that Prdm 4 is found both in the cytosol and the nucleus of HCEC-12 cells (Fig. 3A). Moreover, Prdm 4 expressing HCEC-12 cells also expressed a well-known marker of corneal endothelial cells, the Na^+^/K^+^-ATPase (Fig. 3B). We then investigated Prdm 4 protein expression in primary hCECs and observed that Prdm 4 is co-expressed in hCECs together with the corneal endothelial cell marker, Na^+^/K^+^-ATPase (Fig. 3C). We found that Prdm 4 is expressed at relatively high levels in these cells and localizes to the cytosol and to a lesser extent to the nucleus of primary hCECs (Fig. 3C).

**Fig. 4:**
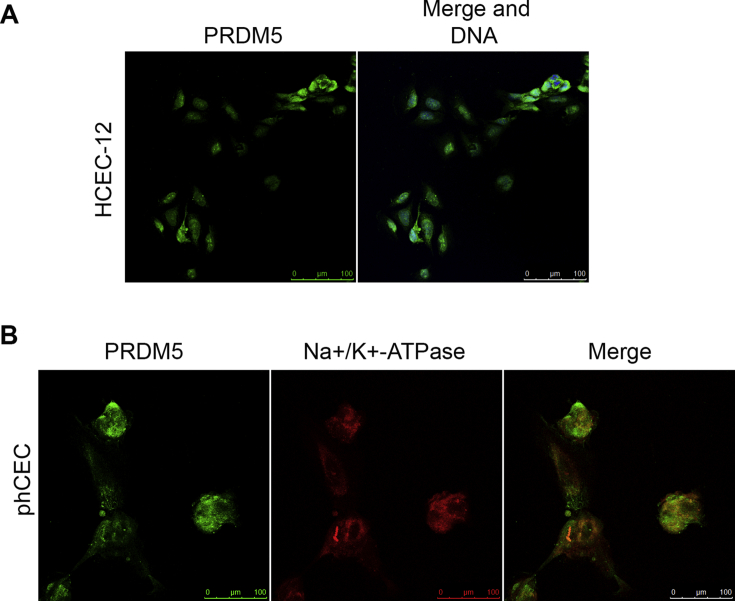
Prdm 5 protein is expressed in HCEC-12 cells and primary hCECs. A) Fluorescent immunolabelling of HCEC-12 cells reveals that Prdm 5 protein (green) is localised to the nucleus and the cytosol of immortalised HCEC-12 cells. B) Prdm 5 protein (green) is expressed in primary hCECs (phCEC) together with the Na^+^/K^+^-ATPase (red). Nuclei are counterstained with Hoechst (blue). (For interpretation of the references to colour in this figure legend, the reader is referred to the web version of this article.)

Immunostaining for HCEC-12 cells and primary hCECs with antibodies against Prdm 5, revealed that the protein is expressed in both cell types (Fig. 4A and B). Similar to Prdm 4, Prdm 5 was found in both the cytosolic and the nuclear compartments of the cells and co-localised with hCECs expressing Na^+^/K^+^-ATPase (Fig. 4B). We conclude, that both, Prdm 4 and Prdm 5 proteins are expressed in primary human corneal endothelial cells as well as the immortalised HCEC-12 cells together with Na^+^/K^+^-ATPase. Moreover, both proteins can be detected in the nucleus and the cytosolic compartments of the cells suggesting that they function in both and that their transport/accumulation in these two compartments may be regulated.

### 3.4 Expression of *Prdm* 4 and *Prdm* 5 proteins in human corneal epithelium, stromal keratocytes and endothelium

**Fig. 5:**
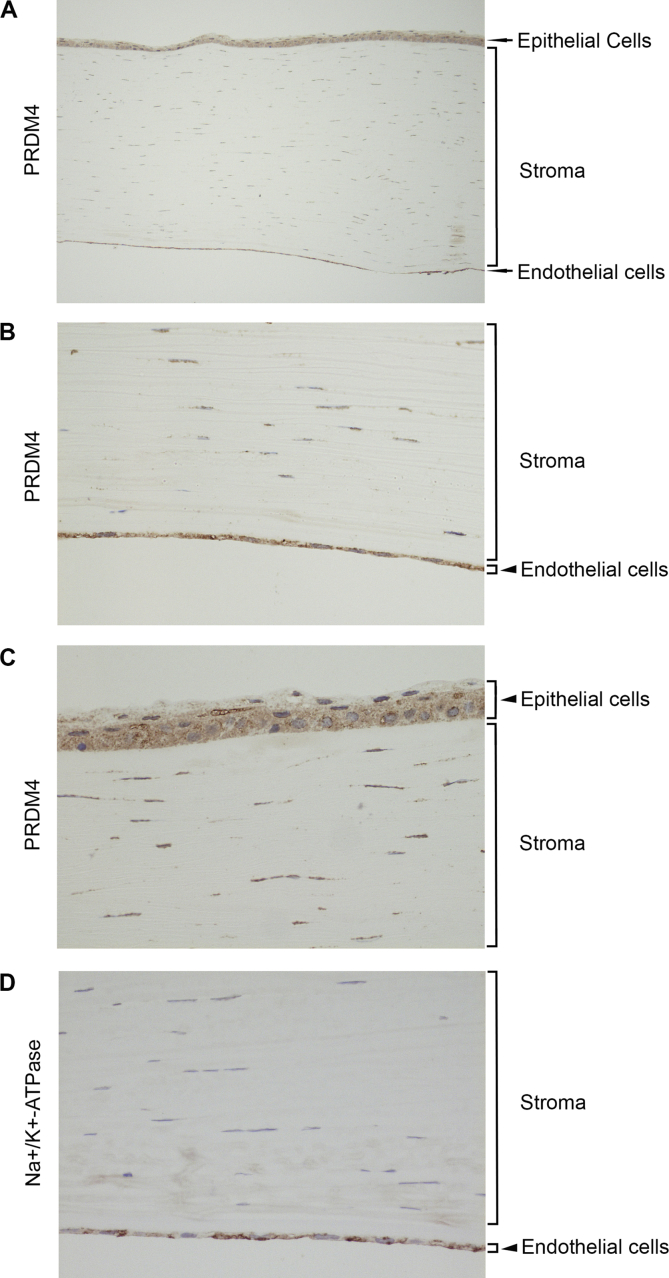
Prdm 4 protein is expressed in the intact cornea. A) Immunolabelling of the intact cornea showing an overview of Prdm 4 protein distribution within the tissue. Prdm 4 (brown staining) is found in the epithelial cells, endothelial cells and keratinocytes of the stroma. B) and C) Higher magnification of the images demonstrating Prdm 4 expression in the endothelial (B) and epithelial (C) cells of the cornea. Prdm 4 immunoreactivity is detected in the cytosol, concentrated at the perinuclear regions and weakly in the nucleus of these cells. D) Endothelial cells of the cornea are immunolabelled with antibodies against the Na^+^/K^+^-ATPase (brown staining), which is found at the membranes of these cells. Tissue sections are counterstained with haematoxylin to visualise nuclei (blue) in all panels. (For interpretation of the references to colour in this figure legend, the reader is referred to the web version of this article.)

**Fig. 6:**
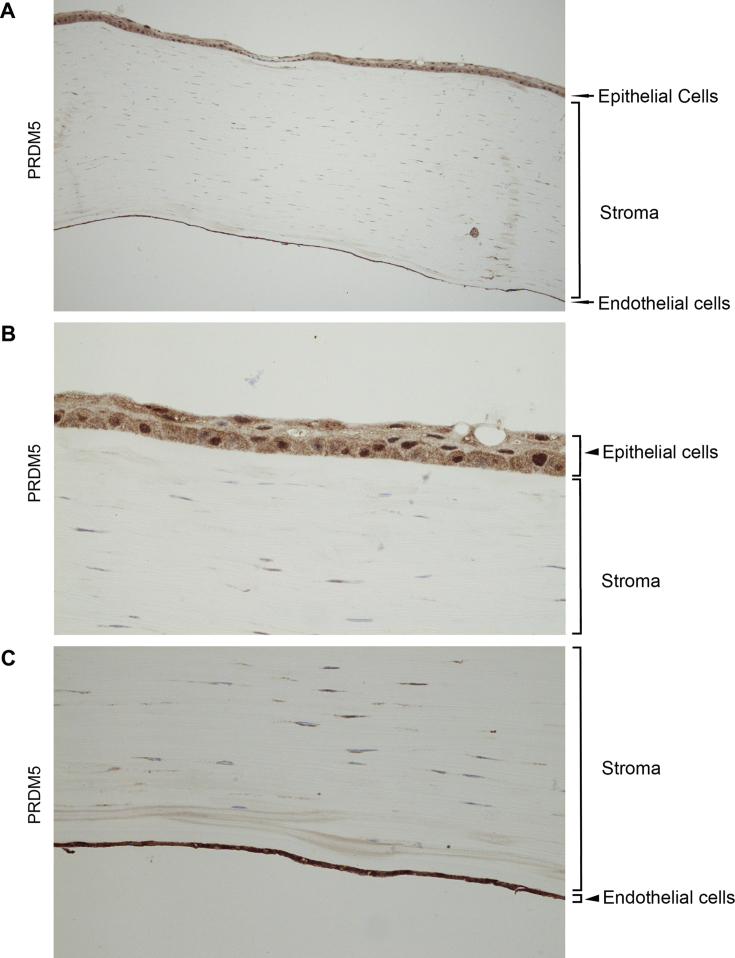
Prdm 5 protein is expressed in the intact cornea. A) Immunolabelling of the intact cornea showing an overview of Prdm 5 protein distribution within the tissue. Prdm 5 (brown staining) is found in the epithelial cells, endothelial cells and keratinocytes of the stroma. B) and C) Higher magnification of the images demonstrating Prdm 5 expression in the epithelial (B) and endothelial (C) cells of the cornea. Prdm 5 exhibits strong immunoreactivity in the nuclei of both cell types and diffuse distribution within the cytosol of the cells. Tissue sections are counterstained with haematoxylin to visualise nuclei (blue) in all panels. (For interpretation of the references to colour in this figure legend, the reader is referred to the web version of this article.)

To assess the tissue distribution of Prdm 4 and Prdm 5, we investigated the distribution of both proteins in intact human corneas. Both proteins were found in the endothelial cells of the cornea as well as the epithelial cell layer and stromal keratocytes (Fig. 5, Fig. 6). Prdm 4 was found evenly distributed between both the cytosolic and the nuclear compartments of the endothelial and epithelial cells, somewhat enriched in perinuclear areas (just outside the nucleus) (Fig. 5 A, B and C), contrasting with the predominantly cell membrane-specific immunoreactivity observed for the endothelial cell marker, Na^+^/K^+^-ATPase (Fig. 5D). Interestingly, it appeared to localise to the nuclei of the stromal cells. Prdm 5 showed a much stronger immunoreactivity in both the endothelial and the epithelial cells compared to the levels detected for Prdm 4 (Fig. 6 A, B and C). Moreover, while Prdm 5 was also found in both cytosolic and nuclear compartments of these cells, it appeared much more concentrated in the nucleus than Prdm 4. Furthermore, no specialised enrichment of Prdm 5 in the perinuclear areas was observed. Within the stromal keratocytes, Prdm 5 also appeared localised to the nucleus and exhibited much weaker staining than in either the epithelial or endothelial cells. The observed patterns of subcellular distribution of both Prdm 4 and Prdm 5 suggest that the functions these proteins have in corneal cells may be distinct.

## 4 Discussion

Corneal endothelial cells are a unique population of cells which function to preserve corneal clarity. CECs are generally known to be post mitotic in their cell cycle with poor proliferative capacity in vivo and in vitro. This offers significant limitations to generate CEC cultures in vitro for cell therapy, but nevertheless shown to be possible in recent studies (Hara Susumu et al., 2014). In our work, we focussed on the identification of the *Prdm* genes, expressed in the cornea and, specifically, in corneal endothelial cells, with a long-term view to begin understanding their roles in CEC differentiation and biology. Prdm family of transcriptional and epigenetic regulators control differentiation and re-programming of a wide variety of cell lineages (Fog et al., 2012, Hohenauer and Moore, 2012, Zannino and Sagerström, 2015, Chi and Cohen, 2016) and we reasoned that they may also play a role in controlling proliferation and differentiation of CECs.

In the first instance we isolated total RNA from whole human corneas and performed RT-PCR to investigate the expression profile of all *Prdm* genes. We identified *Prdm 1, 2, 4, 5* and *10* in the intact healthy corneas. We then investigated the expression patterns of Prdm 4 and 5 proteins in more detail, as these two proteins have been implicated in the control of corneal function, and discuss them further in the context of their relevance to the CEC biology.

Prdm 4 was originally identified in a screen for low affinity neurotrophin receptor, p75NTR, interacting proteins (Chittka and Chao, 1999) and has been shown to function as a transcriptional regulator which recruits chromatin modifying enzymes, such as histone deacetylases (HDACs) (Chittka et al., 2004) and a protein arginine methyltransferase 5 (PRMT5) (Chittka et al., 2012) to control gene expression. *Prdm 4* is broadly expressed in many tissues suggesting an important role for this protein in the control of cellular differentiation and “stemness”, in part through neurotrophin-mediated signalling. This is especially relevant given that both p75NTR and another Nerve Growth Factor receptor, TrkA, have been shown to be expressed in limbal basal epithelial cells of the cornea, the primary site of the limbal stem cells (Qi et al., 2008) and p75NTR is found in the human corneal endothelial progenitor cells where it has been used to isolate and culture potential hCE progenitor cells successfully (Hara Susumu et al., 2014). Intriguingly, mice with a homozygous deletion of *Prdm 4* gene display gender-specific abnormal corneal morphology suggesting that Prdm 4, perhaps downstream of the p75NTR signalling, may be one of the critical determinants of corneal biology (MRC Harwell web reference). In this respect, it is noteworthy that we detected Prdm 4 protein expression both in the epithelial and the endothelial cells of the cornea. Furthermore, our previous observations that Prdm 4 maintains the “stemness” of cortical neural stem cells during their development (Chittka et al., 2012) suggest it may be important for mediating a similar control of developmental fates of hCECs. Taken together, our observations that *Prdm 4* is expressed in the corneal endothelial cells, that it was initially isolated as p75NTR interacting protein and that the mice where *Prdm 4* gene is deleted display abnormalities in corneal morphology, strongly implicate Prdm 4 in critical control of corneal endothelial cell differentiation.

Prdm 5 protein has been implicated in corneal biology as mutations in *Prdm 5* are associated with the BCS and other corneal abnormalities (Burkitt Wright et al., 2011, Aldahmesh et al., 2012, Porter et al., 2015a, Porter et al., 2015b, Micheal et al., 2016a, Micheal et al., 2016b). In part, Prdm 5 controls corneal integrity by regulating expression of extracellular matrix components, such as fibrillar collagens, and loss of Prdm 5 function leads to corneal fragility and rupture often resulting in blindness (Burkitt Wright et al., 2011). Our finding of Prdm 5 expression in both corneal epithelial and endothelial cells is an interesting addition to this reported association.

Prdm 1 has a well characterised function in the control of primordial germ cell lineage (Ohinata et al., 2005, Günesdogan et al., 2014) and B-cell lineage (Savitsky et al., 2007) as well as multiple other cell lineages (Fog et al., 2012, Hohenauer and Moore, 2012), but no information is available about its potential role in corneal development. Prdm 2, also known as RIZ1 (Retinoblastoma-interacting zinc-finger protein 1), is a tumour suppressor which exhibits histone H3 lysine 9 methyltransferase activity (Derunes et al., 2005) and has been shown to be inactivated in a variety of tumours (Liu et al., 2013, Tan et al., 2014, Zhang et al., 2015). Similar to Prdm 1, no information is available about Prdm 2 function in the cornea. Prdm 10, also known as Tristanin, appears to be expressed in the central nervous system and may be involved in the pathogenesis of neuronal storage diseases which lead to mental retardation and early childhood death (Siegel et al., 2002). Similar to Prdm 1 and 2, there is currently no information available about its function in the cornea.

In our analysis of *Prdm* genes expressed in the Descemet membrane/corneal endothelial cells, we noticed that *Prdm 1* mRNA was no longer detectable suggesting that *Prdm 1* expression may not be specific to the corneal endothelial cells. We also detected a very weak *Prdm 11* expression in this sample. We do not know if the expression of *Prdm 11* is induced by the preparation of the DM/CECs or whether the isolated tissue may have been slightly contaminated with cells of other origin. It is noteworthy in this respect that *Prdm 11* mRNA is also detectable in immortalised HCECs, perhaps indicating alterations in expression profiles of *Prdm* genes following ex vivo culture and immortalization. Similar to Prdm 1, 2 and 10, no information is available on the potential function of Prdm 11 in the cornea.

In assessing expression of *Prdm* genes in two cellular systems, primary hCECs established from DM/CECs and in an immortalised cell line, HCEC-12, we discovered that there were some differences in the expression profiles of *Prdm* genes between DM/CECs isolated from the cornea and both of the above cell lines. The most notable difference between the DM/CEC tissue and the established hCECs was expression of *Prdm 8* in the latter cells. Prdm 8 is known to be expressed in the developing mouse retina (Komai et al., 2009) and is important in controlling rod bipolar and type 2 OFF-cone bipolar cell survival as well as amacrine subtype identity (Jung et al., 2015), implicating it in the regulation of specific aspects of eye development and differentiation of specific cell types within the visual system. However, Prdm 8 has not been implicated in the control of corneal function and it is unclear whether primary hCECs we established express it because of culture conditions or perhaps some form of re-programming which may occur during longer-term culturing of cells.

Immortalised HCEC-12 cells showed marked differences in the expression profile of detectable *Prdm* genes compared to those expressed in DM/CECs isolated from intact cornea. In particular, *Prdm 3* (MECOM isoform), *6, 8* and *16* were detected in HCEC-12 cells by RT-PCR in addition to those observed in DM/CECs tissue preparations. Given that HCEC-12 cells are immortalised using the early region of SV40 genome containing the large T antigen and the small t-antigen, they may be re-programmed to express other *Prdm* genes as part of their phenotype. For example, Prdm 3 (Mecom form) has a well-documented role in myeloid leukemogenesis (Juneja et al., 2014) where it appears to control haematopoietic stem cell (HSC) quiescence and survival of primary human leukaemia cells; in this context Prdm 3 is part of the core “stem cell” gene cluster shared by both HSCs and leukaemia stem cells (Eppert et al., 2011). Conceivably, its expression in immortalised HCEC-12 cells reflects their new cellular identity which may upregulate some of the “stem cell” gene cluster genes to enhance the survival of these cells. Prdm 6 has been shown to inhibit endothelial cell proliferation, survival and differentiation (Wu et al., 2008) and its expression in the HCEC-12 may be induced as part of the re-programming of these cells. Prdm 16 is an important regulator of adipose tissue as well as haematopoietic and cardiac tissue (Seale et al., 2007, Kajimura et al., 2009, Arndt et al., 2013, Chi and Cohen, 2016). Further, Prdm 16 has been shown to regulate oxidative stress in both haematopoietic and neural stem cells (Chi and Cohen, 2016) and some of the known chromosomal rearrangements involving *Prdm 16* in humans lead to haematological malignancies (Chi and Cohen, 2016). Therefore, Prdm 16 may also contribute to the re-programmed phenotype of HCEC-12 cells after their immortalization. Of note, *Prdm 1, 2, 3* and *16* genes encode two isoforms, those which contain the PR/SET domain and those which lack it, and the biological roles of these two isoforms in controlling cellular proliferation are opposite, with the PR/SET-containing isoforms being anti-proliferative and the PR/SET-lacking isoforms supporting cellular proliferation in a variety of cells (Di Zazzo et al., 2013). Therefore, it will be important to investigate the distribution of different isoforms of detected *Prdm* genes in various cell lines as well as in normal cornea and primary hCECs in the future in order to understand their potential roles in controlling CEC proliferation and differentiation.

In this report we focussed our attention on the characterisation of expression of *Prdm* genes in human cornea with the special emphasis on the corneal endothelial cells with the view of identifying those *Prdm* genes which may be important for CEC biology. Of the *Prdm* genes identified in our screen, we further analysed expression of Prdm 4 and 5 proteins in intact corneas as well as primary hCECs and HCEC-12 cell line. We found that Prdm 4 and 5 are expressed in both corneal endothelial and epithelial cells with slightly different subcellular distribution of these two proteins within the above cells. The remaining *Prdm* genes identified in this screen will warrant further study to understand their contribution to the biology of CECs. We focussed on *Prdm 4* and *5* genes in part because there is evidence which implicates both of these proteins in corneal biology. The role of Prdm 5 is by far better understood, while Prdm 4 is clearly a novel candidate with potentially important roles in corneal biology. Indeed, it is especially intriguing, given that *Prdm 4* was initially cloned in a screen for p75NTR interacting proteins (Chittka and Chao, 1999), as p75NTR has been used successfully to enrich for corneal endothelial precursor cells (Hara Susumu et al., 2014). It will be important in the future to investigate the precise role of Prdm 4 in corneal biology and the biology of corneal endothelial cells with a view of potentially using it as a marker in conjunction with p75NTR.

## 5 Conclusions

We present evidence that *Prdm 2, 4, 5* and *10* genes are expressed in hCECs and in the whole human cornea. Moreover, Prdm 4 and 5 proteins are found in the CECs of the cornea where they are distributed both in the nucleus and the cytosol. Identification of these in part novel genes, which are important in controlling cellular proliferation and differentiation, adds to the repertoire of potentially important players in helping the generation of CECs for therapeutic purposes in future studies.

## Funding sources

AC and CG were supported by the BBSRC grant BB/J006602/1. KR and MR were supported by a PhD studentship from Anglia Ruskin University and the Vision and Eye Research Unit REF2014 QR fund. The funders did not participate in the work or data processing.
